# Supplementary figures and images for: Neutrophils Encompass a Regulatory Subset Suppressing T Cells in Apparently Healthy Cattle and Mice
Source: Front Immunol. 2021 Feb 26;12:625244. doi: 10.3389/fimmu.2021.625244 (PMC7952614; doi:10.3389/fimmu.2021.625244)

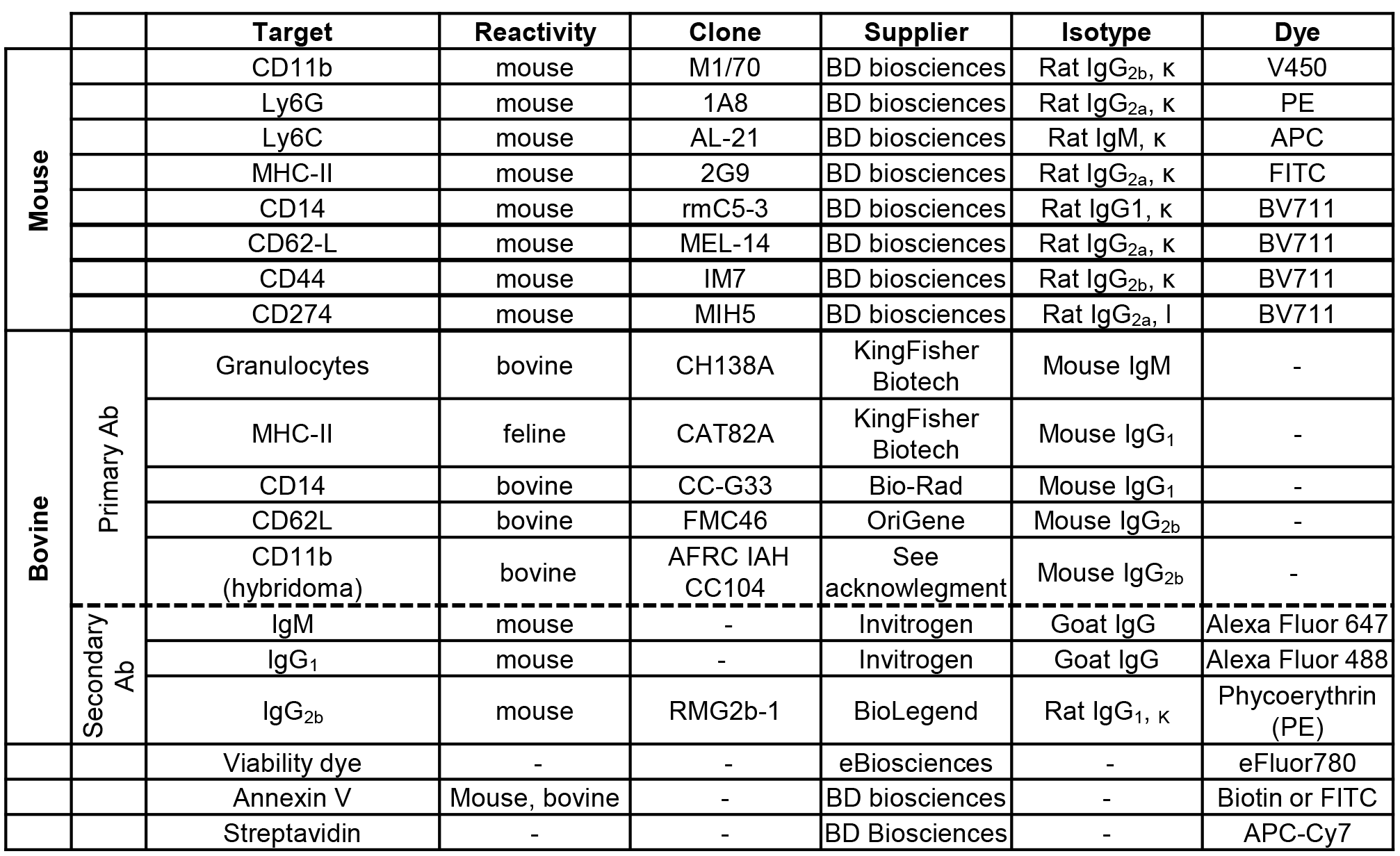

Supplement: Supplementary Table 1 — Antibodies used in the study. The specificity, origin, clone number and commercial provenance of all antibodies used in the study are listed. [file Image_6.tif]

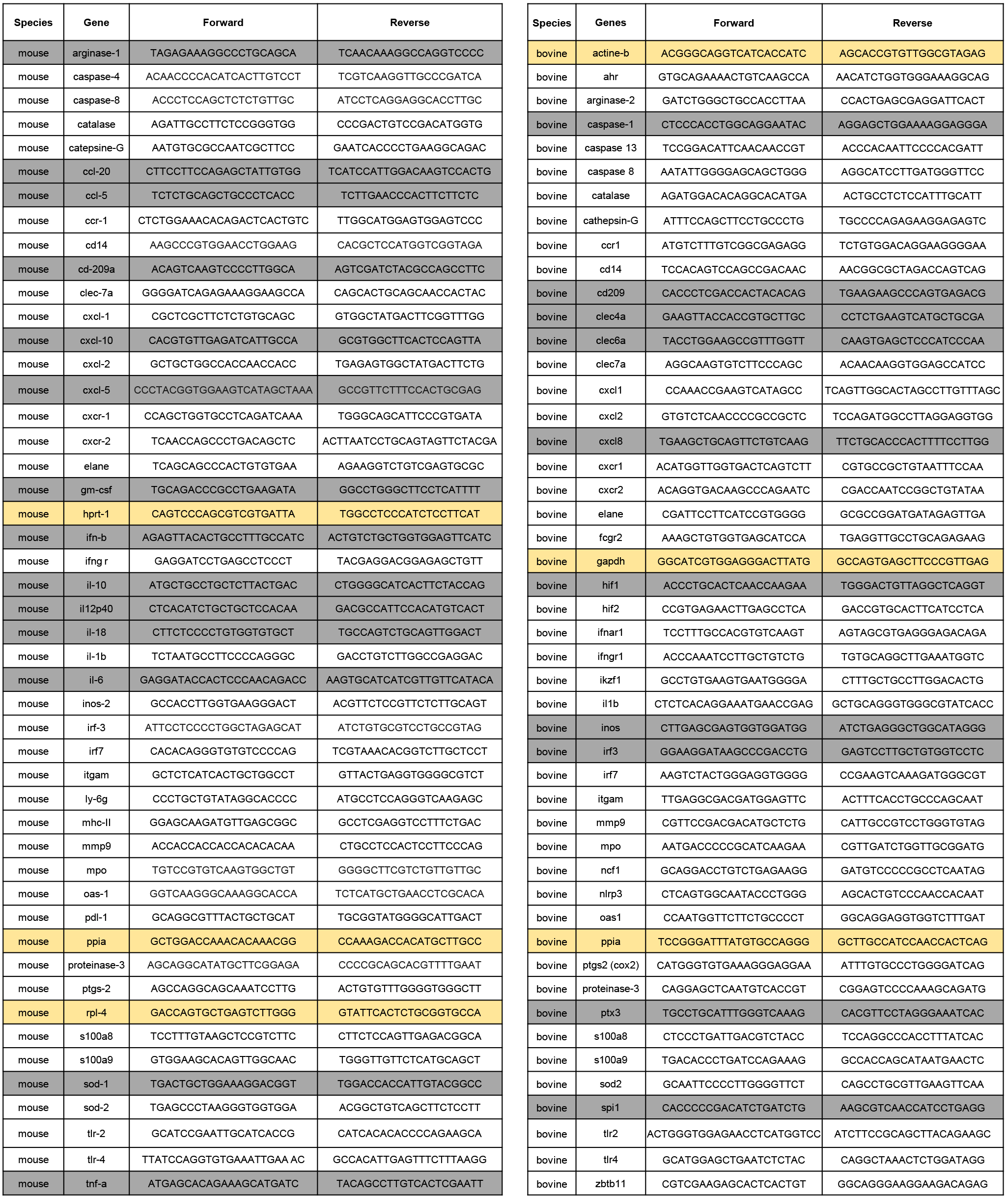

Supplement: Supplementary Table 2 — Sequences of primers used in this study. Primers were designed using Geneious software, in intron-spanning regions when possible. The annealing temperature was set at 60 and 62°C for bovine and mouse samples respectively. Housekeeping genes used as the reference to calculate ΔCT for each species are indicated in the yellow boxes and weakly expressed genes that were removed from the Principal Component Analysis presented in Figure 4 are indicated in the grey boxes. [file Image_7.tif]

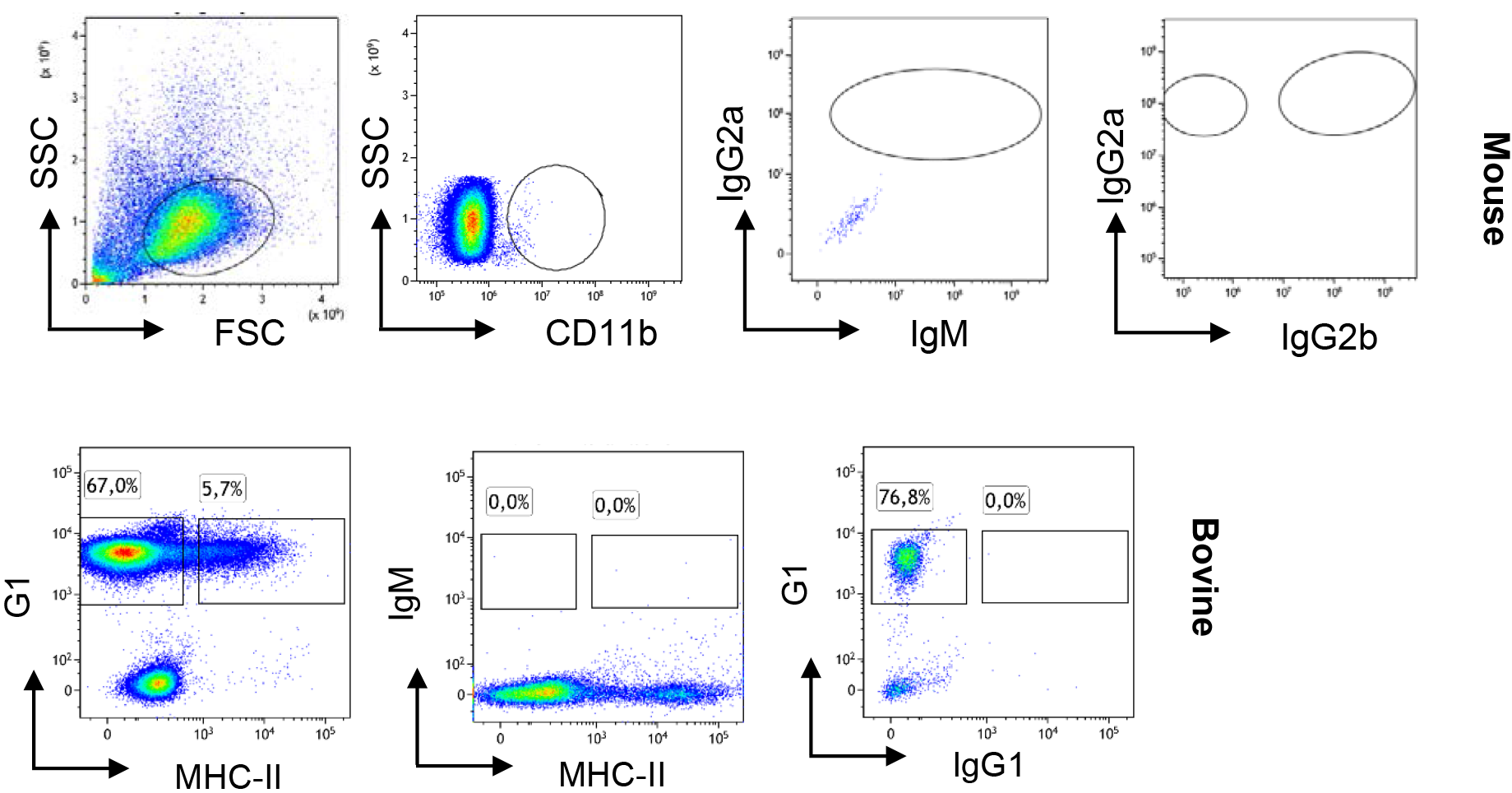

Supplement: Supplementary Figure 1 — Isotype controls for neutrophil diversity analysis in mouse bone marrow and cattle blood. Mouse and bovine neutrophils were labelled as described in Figure 2. Appropriate isotype controls for all antibodies in each experiment were used to correctly set the analysis and sorting gates. Dot plots from one representative animal are depicted (3 independent experiments, n=4 mice, n=6 for bovine). [file Image_1.tif]

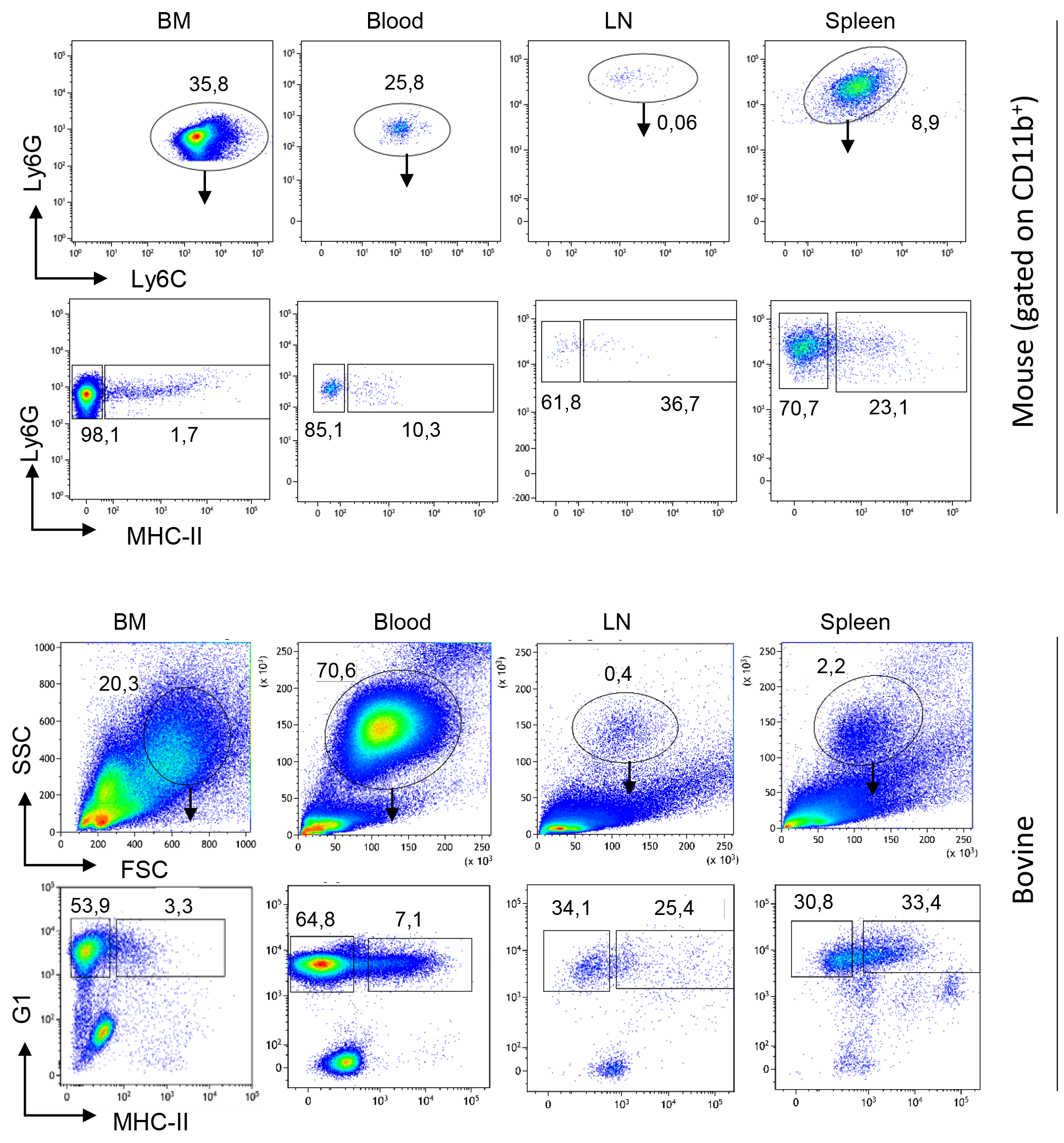

Supplement: Supplementary Figure 2 — Gating strategy for neutrophil diversity analysis in all organs. Mouse and bovine neutrophils were labelled as described in Figure 2. Similar procedures were set up with isotype controls for all antibodies in each experiment to correctly set the analysis gates. Dot plots from one representative animal are depicted (3 independent experiments, n=4 mice, n=6 for bovine). [file Image_2.tif]

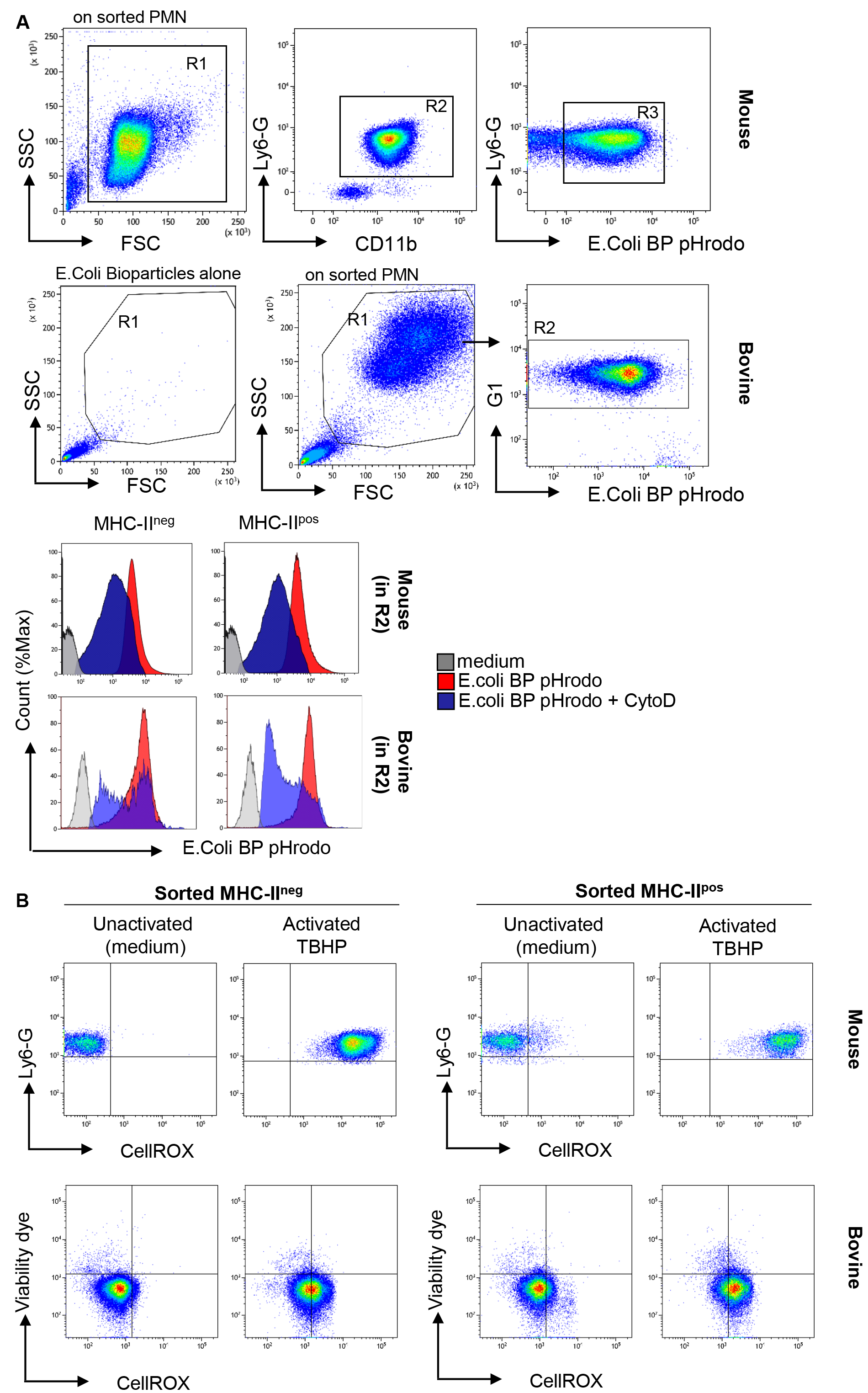

Supplement: Supplementary Figure 3 — Gating strategy for analysis of phagocytosis and ROS production by neutrophils. Neutrophils were labelled and sorted as described in Figures 2 and S1. (A) After purification by cell sorting from the BM (mouse) or blood (bovine) phagocytosis by MHC-IIpos or MHC-IIneg neutrophils was assessed using pHrodo E.coli bioparticles with or without previous treatment with cytochalasin D. Dot plots from one representative animal are depicted (3 independent experiments, n=3 pool of 10 mice, n=3 for bovine). (B) Oxidative stress was measured in MHC-IIpos and MHC-IIneg sorted neutrophils using the CellROX Orange probe that reacts with all ROS species. Cells were activated with TBHP or incubated with medium alone and levels of ROS were measured by flow cytometry among the live cells (unstained with eFluor780 viability dye). Dot plots from one representative animal are depicted (3 and 4 independent experiments for mice and cattle respectively, n=3 pool of 10 mice, n=5 for bovine). [file Image_3.tif]

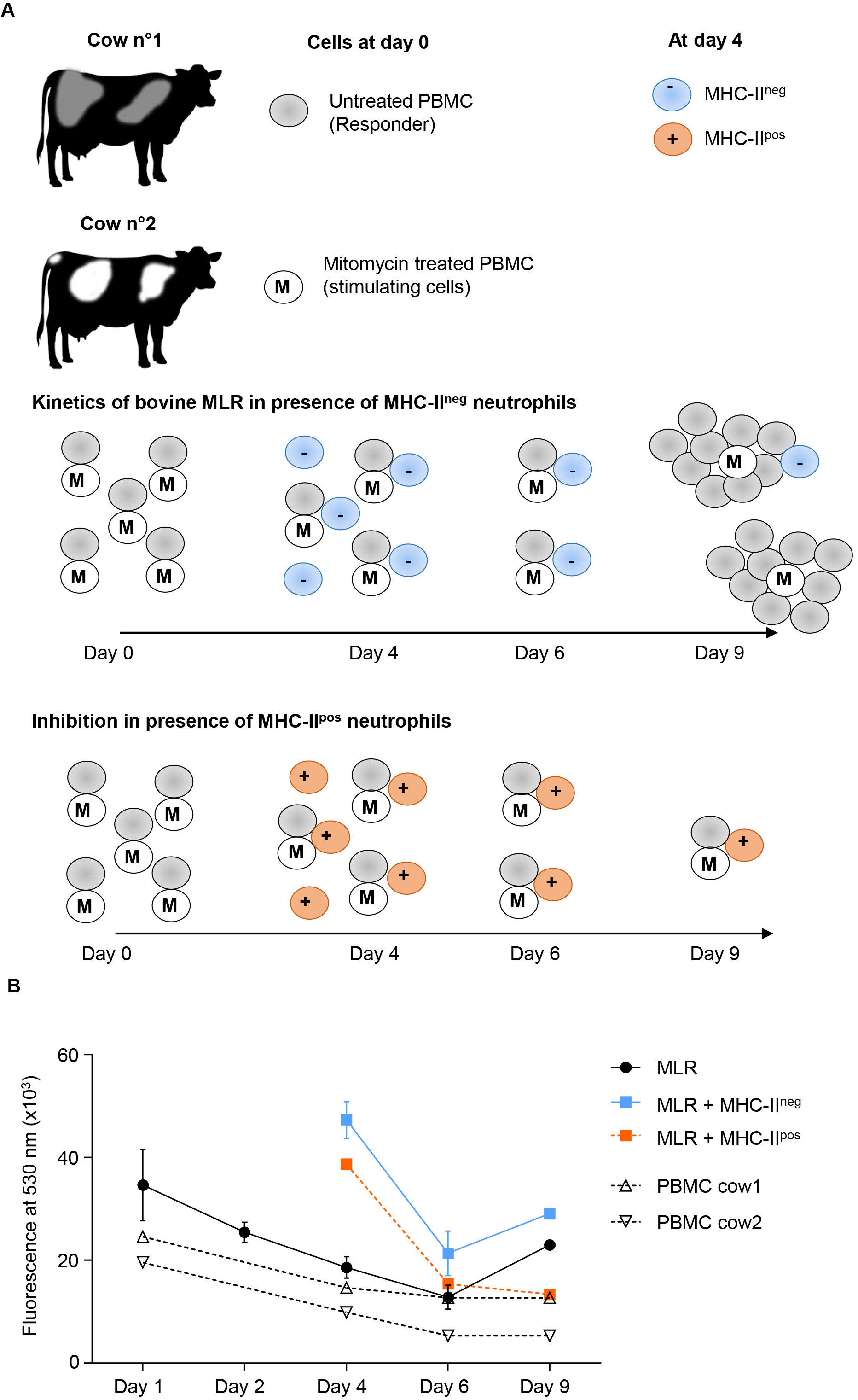

Supplement: Supplementary Figure 4 — Kinetics of bovine Mixed Leukocyte Reaction and analysis of neutrophil suppressive activity. (A) PBMCs from the responder animal were isolated and left untreated, while PBMCs from the stimulating animal were incubated with mitomycin C to block their proliferation. PBMCs from the two cows were incubated at ratio of 1:1. Sorted MHC-IIpos or MHC-IIneg neutrophils from the responder animal were added to the reaction at day 4. (B) DNA was quantified at different time points with CyQUANT Cell Proliferation Assay tests according to manufacturer’s instruction and fluorescence was read at 530nm. DNA extracted from PBMCs cultivated separately decreased along the assay indicated the absence of proliferation (dotted lines). In the MLR reaction, while DNA content declined between day 1 and 6, PBMCs proliferation could be measured between day 6 and 9 (black). The effect of adding sorted MHC-IIneg neutrophils (blue) or MHC-IIpos neutrophils (orange) to the proliferating cells could then be measured. One representative experiment is shown and data represent the mean ± SEM of technical triplicates. Four independent experiments were conducted with different pairs of cows. [file Image_4.tif]

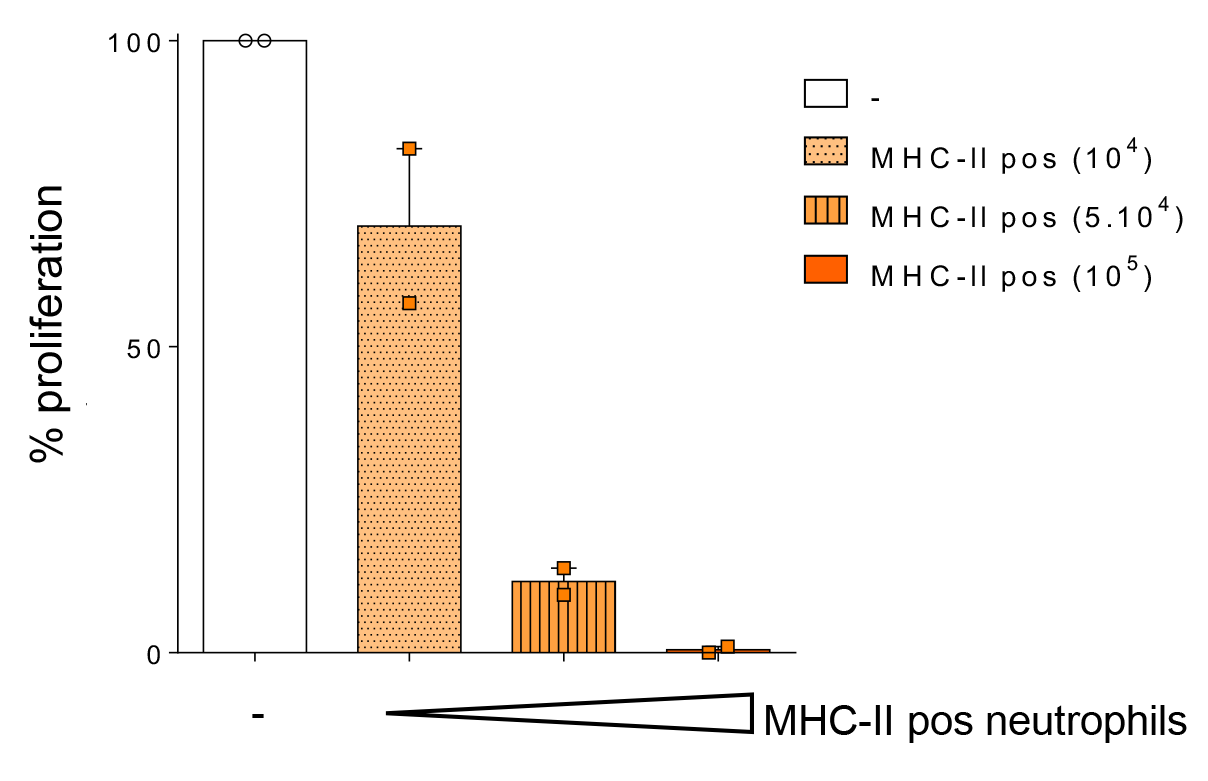

Supplement: Supplementary Figure 5 — Bovine MHC-IIpos neutrophil suppressive activity is dose-dependent. The suppressive assay was performed as described in Figures 6 and S4. At day 4, 1x10^5^, 5x10^4^ or 1x10^4^ purified MHC-IIpos neutrophils from the responder animal were added to the MLR reaction. One experiment was performed (technical duplicates are depicted). [file Image_5.tif]
